# Dietary Assessment Methods in Military and Veteran Populations: A Scoping Review

**DOI:** 10.3390/nu12030769

**Published:** 2020-03-14

**Authors:** Rebecca A. Collins, Bradley Baker, Daisy H. Coyle, Megan E. Rollo, Tracy L. Burrows

**Affiliations:** 1 School of Health Sciences, Faculty of Health and Medicine, University of Newcastle, Callaghan 2308, Australia; Rebecca.Collins10@uon.edu.au (R.A.C.); Megan.Rollo@newcastle.edu.au (M.E.R.); 2 Priority Research Centre for Physical Activity and Nutrition, University of Newcastle, Callaghan 2308, Australia; 3 Food and Nutrition, Land Division, Defence Science and Technology Group, Scottsdale 7260, Australia; bradley.baker2@dst.defence.gov.au; 4 The George Institute for Global Health, University of New South Wales, Sydney 2042, Australia

**Keywords:** scoping review, defense, military, diet, dietary assessment

## Abstract

Optimal dietary intake is important for the health and physical performance of military personnel. For military veterans, the complex nature of transition into civilian life and sub-optimal dietary intake is a leading contributor to the increased burden of disease. A scoping review was undertaken to determine what is known about the assessment and reporting of dietary intakes within both military and veteran populations. In addition, this review determines if studies reporting on the dietary intake of military personnel or veterans include comparisons with dietary guidelines. Six databases were searched to identify papers published from the database inception to April 2019. Observational and intervention studies were searched to identify if they assessed and reported whole dietary intake data, reported data exclusively for a military or veteran population, and included only healthy populations. A total of 89 studies were included. The majority of studies used one dietary assessment method (*n* = 76, 85%) with fewer using multiple methods (*n* = 13, 15%). The most frequent methodology used was food frequency questionnaires (FFQ) (*n* = 40, 45%) followed by 24-hour recalls (*n* = 8, 9%) and food records (*n* = 8, 9%). The main dietary outcomes reported were macronutrients: carbohydrate, protein, fat, and alcohol (*n* = 66, 74%) with total energy intake reported in *n* = 59 (66%). Fifty four (61%) studies reported a comparison with country-specific dietary guidelines and 14 (16%) reported a comparison with the country-specific military guidelines. In conclusion, dietary intake in military settings is most commonly assessed via FFQs and 24-hour recalls. Dietary intake reporting is mainly focused around intakes of energy and macronutrients. Most studies compare against dietary guidelines, however, comparison to specific military dietary guidelines is minimal.

## 1. Introduction

Optimal dietary intake is a crucial component of a military environment due to the high physical and mental demands placed on personnel [1,2,3,4]. Research to date has shown that the health, fitness and performance of military personnel is highly reliant on their nutritional status during training, and, therefore, their readiness to be deployed can be negatively affected by suboptimal nutrient intakes [2,3,4,5]. This is particularly relevant to service members in combat roles when on base preparing/training for field deployment [6]. To support their training, a focus on a healthy and optimal diet is essential for facilitating training adaptations, recovery, maintenance of health, and career progression within the military. Optimal nutrition is also important for reducing the risk of long-term health conditions such as obesity and cardiovascular disease [7,8]. This is not only pertinent to personnel actively serving in the military, but also to military veterans. As such, it is important to also consider the dietary intakes of the veteran community when assessing dietary factors that are linked to long-term health conditions.

The dietary requirements of active military personnel vary according to their occupation and work environment due to the differing physical and mental workloads across the vast range of roles and environments in which military personnel operate [6,9,10]. Considering this, in 2001, the United States of America (USA) military developed specific dietary guidelines, known as the Military Dietary Reference Intakes (MDRIs) [11,12]. The MDRIs incorporate aspects of the Dietary Guidelines [13] for Americans [1]. The guidelines also consider context specific factors such as the physical activity levels of military personnel and environmental elements, such as extremely hot desert environments [14]. Similarly, in Australia, the Australian Defence Force (ADF) also follows military specific dietary guidelines [15], which are based on the Australian Nutrient Reference Values (NRVs) [6]. Similar to in the USA, the guidelines take into account the various occupations and scenarios encountered by military personnel and their often increased macronutrients requirements. These are divided into five activity categories; those undertaking moderate to high physical activity and those in more sedentary roles [15]. The Nordic countries (Denmark, Finland, Iceland, Norway, Sweden) construct their military diets from the Nordic Nutrition Recommendations (NNR) [16], with the North Atlantic Treaty Organisation (NATO) ensuring military operations are meeting the mandatory food standards [16]. The standards include guidelines around the day-to-day nutrition provisions within the military environment [16].

The military environment is distinctive and some personnel may be required to shift their usual dietary intake between fresh foods to combat rations, which can be accompanied by variations in daily energy expenditure due to the impacts of training [17]. For example, the garrison environment, when compared to field work, often has a lessened physical impact on personnel with longer hours of recuperation periods and planned meal times [17,18]. Within the same garrison, the type of personnel must also be taken into account, for example, special forces personnel are likely to have much higher energy demands than personnel completing desk-based tasks [11,19]. In contrast, veterans acquire most of their daily dietary intake in a home/community environment similar to the general population [6,20]. Numerous studies have reported on the dietary intakes within military and veteran populations [21,22,23,24,25]. However, the data from these studies have not been systematically reviewed and synthesised for a broad range of military and veteran settings, with the exception of where combat rations are consumed for active military personnel [17]. Considering dietary intake in environments where combat rations are not used is more indicative of the habitual diets of active military personnel, it is important that the scope of the evidence is explored.

The purpose of this scoping review was to investigate the use of dietary intake measures of whole diet in military and veteran populations. The primary question was to determine what is known about the assessment and reporting of whole dietary intakes in individuals within military and veteran populations, from a broad range of settings. The secondary question was to evaluate whether assessment and reporting of whole dietary intake of individuals in the military include comparison with current dietary guidelines. These questions were selected to ensure a broad scope of literature was captured [26].

## 2. Methods

A scoping review was chosen to be undertaken for this topic to systematically synthesise the main sources of whole dietary intake data collected within military or military veteran populations. Scoping reviews assist in outlining the current evidence and possible evidence gaps in areas of research. This scoping review adheres to the PRISMA Extension for Scoping Reviews Checklist (PRISMA-ScR Checklist) [27].

### 2.1. Search Strategy and Study Selection

The search strategy and databases were selected in consultation with a research librarian and six databases were searched—CINHAL, Cochrane, EMBASE, Medline, Proquest (Military Database), and Scopus. The search strategy consisted of associated terms in term groups; diet and military (Table 1) and included English language studies published from database inception until April 2019. The reference lists of key papers were also searched. The review included all study designs, excluding narrative reviews, case report/series, commentaries, editorials, Letters to the Editor, theses and conference proceedings.

Studies were included if they assessed and reported whole dietary intake data such as food groups, macronutrients and/or micronutrients, reported data exclusively for populations of military personnel or military veterans and included only healthy populations, meaning those personnel without specific disease states (e.g., sickle-cell anaemia, eosonphillic eosophogitis). Studies that included individuals with those conditions were excluded from the review. Studies that reported on dietary intake from supplements Tables S1 and S2 as a primary outcome, or only a single aspect of diet, such as only energy intake or vitamin D intake, were also excluded. The decision to exclude these studies was made due to interest in dietary assessment methods of whole diet, and therefore studies not reporting on multi-nutrient intake were excluded.

### 2.2. Study Selection

**Figure 1:**
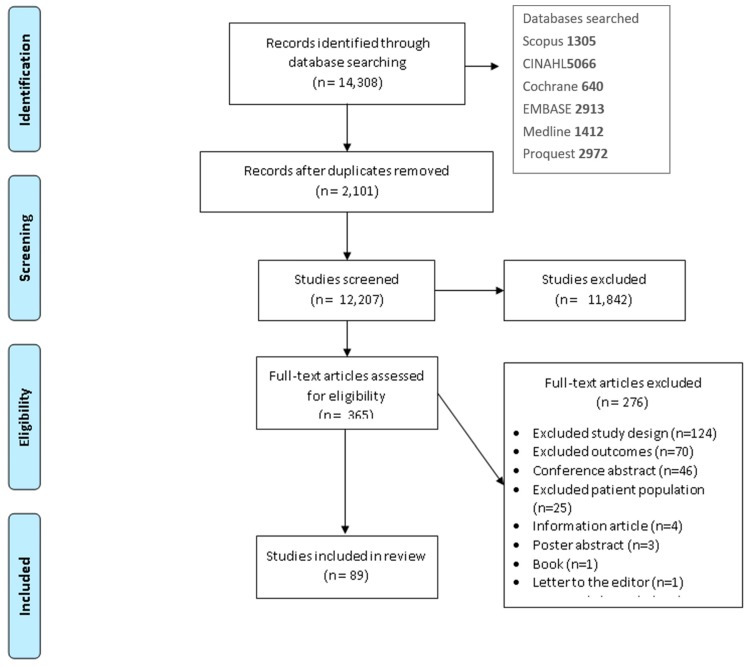
Search strategy process.

After removal of duplicates (Figure 1), two researchers (RC, TB) conducted title and abstract screening. Regular discussions were had between researchers to ensure consistency was being applied to the screening. Conflicts between searchers were removed by a third party (MR) was consulted and any discrepancies were resolved. A full-text review (*n* = 329) was then completed by two researchers (RC, DC, BB), with any conflicts reviewed by a third researcher (TB).

### 2.3. Data Charting

Data were extracted and charted using a data extraction table designed in consultation with all authors. This was initially piloted with 5% of studies to ensure all information was being collected consistently. The extraction table was modified after piloting to include both the main outcomes of the study and the reported outcomes specific to nutrition as it was noted that the main outcomes and nutrition outcomes were often separate. Two researchers conducted the data extraction (RC, DC) which was then checked by a third researcher (BB) for consistency. Study description and outcomes can be viewed in Table 2. For the purpose of this review studies were classified by dietary assessment method (e.g., food frequency questionnaire (FFQ), 24-hour recall, food records). Studies were defined as multi-method if they included more than one form of dietary assessment method, such as a FFQ with food records, and were classified as validated, if the tool utilised in the study was a standardised measure i.e., diet history or direct observation, or referenced a method validation paper. Methods employed were first checked for validation within a general population, followed by validation within military populations. Studies were classified on the number of dietary intake outcomes reported (i.e., energy, fat, protein etc.) and were categorised into one of three groups: less than five outcomes, five to 10 outcomes, or greater than 10 outcomes.

## 3. Results

### 3.1. Search Results

The search strategy identified 11,567 citations (Figure 1). After removal of duplicates, 9920 citations were screened. Following title and abstract review, 311 studies underwent full text screening, of which 233 studies were excluded (Figure 1). The main reasons for study exclusions were; study design (*n* = 106, 44%) e.g., if the study did not include quantitative dietary intake measures, or study outcomes (*n* = 52, 21%) e.g., if the study reported energy expenditure outcomes as opposed to dietary energy or nutrient intake outcomes

### 3.2. Study Designs

Of the 89 studies included, 68 were observational studies including 50 (56%) cross-sectional studies [6,8,22,23,24,28,30,31,35,37,41,42,43,45,46,50,52,55,57,58,59,60,61,63,64,66,68,70,72,73,74,75,76,77,78,79,80,81,82,83,85,87,89,90,92,95,96,98,99,101,106], 10 (11%) prospective cohort studies [2,20,34,37,44,47,91,93,102], two (2%) prospective longitudinal cohort studies [97,105], and two (2%) retrospective cohort study [25,100]. There was one (1%) of each study design for longitudinal cohort [21], validation study [71], cohort study [88], and retrospective cross-sectional study [29]. Twenty-one of the studies were interventions including eight (9%) randomised control trials [7,36,39,51,53,89,94,103], four (4%) pre-post design studies [21,48,49,62], three (3%) non-randomised control trials [40,84,86], two (2%) case-control trials [65,104], and two (2%) non-controlled trial [32,56]. There was one (1%) of each study design for retrospective case-control trial [38] and randomised trial [54].

### 3.3. Study Location

The year of publication of studies ranged from 1973–2019, with 63 (71%) of the studies being published from 2000 onwards [2,6,7,8,20,21,22,23,24,25,29,30,32,34,35,36,37,39,40,43,46,47,48,51,53,56,62,63,64,65,66,70,71,72,73,74,75,76,77,78,81,82,84,85,86,88,89,90,92,94,96,97,98,99,100,101,102,103,104,105]. The continent most frequently studying the dietary intake of their military and veterans was North America. All North American sudies were conducted in the USA, with almost two thirds (*n* = 57, 64%) of included studies originating from this country [6,7,20,24,25,29,30,32,36,37,38,39,40,41,42,43,46,49,51,52,53,54,55,58,59,60,62,63,64,65,66,68,74,77,78,79,80,82,83,88,89,91,92,93,94,95,97,98,99,100,101,102,103,104,105,106]. The remaining studies were carried out in Europe including Belgium (*n* = 6, 7%) [22,70,71,72,73], Finland (*n* = 3, 3%) [21,34], the United Kingdom (*n* = 3, 3%) [28,45,48], Norway (*n* = 3, 3%) [62,94,96], Italy (*n* = 1, 1%) [31], Greece [23], and France (*n* = 1, 1%) [50]. Asia included studies in Israel (*n* = 4, 4%) [2,47,56,81], Iran (*n* = 2, 2%) [76,90] Malaysia (*n* = 1, 1%) [57], and Japan (*n* = 1, 1%) [61]. Two studies were undertaken in Australia (2%) [44,96]. Africa included studies from Cameroon (*n* = 1, 1%) [75], and South Africa (*n* = 1, 1%) [87]. In South America two studies were undertaken in Brazil (2%) [8,35].

### 3.4. Participant Characteristics

The participant numbers included in the 89 studies ranged from 16 t15,747 personnel, with the average number of personnel studied being 218. One study did not report on participant numbers [45]. Forty-five of the 89 studies included male personnel (51%) [2,20,21,23,28,31,34,35,37,39,41,42,44,48,55,58,61,66,70,71,72,73,76,77,79,80,81,82,83,84,85,86,87,90,91,93,94,97,100,102,104,105,106], and approximately a third of the other studies included both male and female personnel (*n* = 35, 39%) [6,7,8,22,24,25,30,37,40,45,46,47,49,51,52,53,54,60,62,63,64,65,68,74,75,78,88,89,92,95,96,98,99,101,103]. Female personnel were exclusively studied in five studies (6%) [29,36,38,56,59], and four studies (5%) did not report on the gender of the personnel included [32,43,50,57]. Research into female military personnel increased post-2000 with 31% (*n* = 28) [6,7,8,22,24,25,30,37,40,46,47,51,53,62,63,64,65,74,75,78,88,89,92,96,98,99,101,103] of studies reporting on outcomes for male and female personnel from 2000–2019 and 3% (*n* = 3) [29,36,56] reporting solely on female personnel from 2000–2016. From 2017–2019 there have been no studies that have included exclusive female populations.

The military population most frequently studied, in descending order, was the army with *n* = 48 (54%) [2,6,7,21,22,23,24,29,30,31,32,34,35,37,38,39,40,43,44,45,46,47,48,50,51,52,53,56,57,58,59,60,61,62,66,70,71,72,73,76,77,82,84,88,89], followed by veterans *n* = 18 (20%) [20,90,91,92,93,94,95,96,97,98,99,100,101,102,103,104,105,106], navy *n* = 8 (9%) [28,42,64,65,68,79,80,83], air force *n* = 8 (9%) [8,36,41,49,54,55,81,87], and National Guard *n* = 2 (2%) [85,86], and five (6%) studies included a combination of different military populations [25,63,74,75,78]. One of the most frequently studied sub-groups of the army are those undertaking basic training or cadet training (*n* = 15/48, 31%) [2,23,31,44,45,52,53,60,62,63,66,84,85,86,88].

### 3.5. Intervention Studies

There were 15 (17%) [7,21,32,36,38,39,49,51,53,54,56,84,89,103] intervention studies of which five included a follow-up component [56,84,86,89,103] ranging from 10 weeks to one year. The population types that included intervention studies were army (*n* = 9) [7,21,32,38,51,53,56,84,89], air force (*n* = 3) [36,49,54], veterans (*n* = 1) [103], National Guard (*n* = 1) [86], and Navy Marines (*n* = 1) [39]. The most commonly studied type of military personnel in included studies were soldiers (*n* = 28, 31%) [6,7,22,24,29,32,35,37,38,45,46,48,50,51,53,56,57,59,62,70,71,72,73,74,76,88] followed by veterans (*n* = 14, 16%) [20,94,95,96,97,98,99,100,101,102,103,104,105,106], and military new recruits (*n* = 9, 10%) [2,23,44,49,63,66,84,85,86].

### 3.6. Dietary Assessment Methods

A range of dietary intake methodologies were used with studies incorporating one dietary assessment method (*n* = 76, 85%) [2,6,7,8,20,22,23,24,25,28,29,32,34,35,36,37,38,39,40,42,43,44,45,46,47,48,49,50,51,53,55,56,57,58,59,60,61,62,63,64,65,66,70,72,73,74,75,76,77,78,79,80,81,82,83,84,85,86,87,88,89,90,91,92,94,97,98,99,100,101,102,103,104,105,106] or multiple methods (*n* = 13, 15%) [21,30,31,37,41,52,54,68,71,93,95,96].

#### 3.6.1. Food Frequency Questionnaires (FFQs)

The most frequent methodology used in included studies was FFQs (*n* = 40, 45%) [2,6,20,21,37,38,41,43,44,47,51,53,56,62,63,64,65,70,71,72,73,74,75,76,78,90,93,96,97,98,99,100,101,102,103,104,105,106] ranging in length from eight to 150 items and administered from once per study up to multiple over a 17-year period. The personnel types to commonly use the FFQ for dietary intake collection were army (*n* = 23/40, 58%) [2,6,21,37,38,43,44,47,51,53,56,62,63,70,71,72,73,74,75,76,78] and veterans (*n* = 14, 35%) [20,90,93,96,97,98,99,100,101,102,103,104,105,106].

#### 3.6.2. Twenty Four-Hour Recalls

The studies that used 24-hour recall (*n* = 8, 9%) [30,34,41,77,81,93,94,95] varied in the number of times administered. Four studies administered a 24-hour recall once throughout the study [30,77,81,95], one study administered two 24-hour recalls at two time points [36], one study the method was administered twice in the main group and two to three times in a subset of participants [94], another had all participants undertake a 24-hour recall three times with a subset undertaking a further three recalls [93]. One study administered four 24-hour recalls [41]. The use of a multiple-pass dietary recall (MPR) approach, within the administration of 24-hour recall, was only reported in one study [77].

#### 3.6.3. Food Records

Food records (*n* = 8, 9%) [8,34,48,52,68,84,85,86] ranging in duration including three days (*n* = 1) [8], four days (*n* = 4) [48,84,85,86], seven days (*n* = 1) [52], and 14 days (*n* = 1) [68]. One study consisting of two sub-studies required one three-day food record and one four-day food record [34]. Four studies were identified as using biomarkers to determine dietary intake [37,53,64,93].

### 3.7. Validity of Dietary Assessment Methods

Of the dietary intake methodologies used, 66 (74%) [2,6,7,8,20,21,24,30,31,32,35,37,38,39,40,43,46,47,48,49,50,51,53,54,56,57,58,59,60,62,63,64,65,68,70,71,72,73,74,75,76,77,78,80,81,82,84,85,86,88,91,92,93,97,98,99,100,101,102,103,104,105,106] were reported as validated methods, including direct observation, 24-hour recall and FFQs or well recognised methods including, dietary records, and diet history [107,108]. Only two studies (2%) were validated specifically for military populations [58,59]. Twenty three studies (26%) did not report validatio [22,23,25,28,29,34,36,41,42,44,45,52,55,61,66,79,83,87,89,90,94,95,96].

### 3.8. Dietary Outcomes

Of the 89 studies, 13 (15%) reported less than five dietary outcomes [21,36,46,48,57,59,72,84,92,95,98,106], 35 (39%) reported five to 10 [8,20,22,23,24,25,31,32,35,37,43,45,47,49,50,52,53,54,55,58,64,65,66,68,73,77,78,85,86,88,89,94,101,102,104], and 41 (46%) reported more than 10 dietary outcomes, which were mostly nutrient profiles [2,6,7,28,29,30,34,37,38,39,40,41,42,44,51,56,60,61,62,63,70,71,72,74,75,76,79,80,81,82,83,87,90,91,93,96,97,99,100,103,105] and less often food based outcomes. Seventy three of 89 studies (82%) [2,6,7,8,20,21,22,23,24,25,29,30,31,32,34,35,36,37,38,39,40,42,43,44,46,47,48,49,51,52,53,54,55,56,57,59,61,63,64,65,66,70,72,73,74,75,76,77,79,81,82,83,85,88,89,90,92,93,94,95,96,97,98,99,100,101,102,103,104,105,106] assessed anthropometric data in addition to dietary assessment. The most commonly used anthropometric assessment was body mass index (BMI) (*n* = 60, 67%) [2,6,7,8,20,21,22,23,24,25,29,30,31,32,34,35,37,38,40,42,44,46,47,49,51,53,54,56,57,61,63,64,65,70,72,73,74,75,76,81,85,88,89,90,92,93,94,96,97,98,99,100,101,102,103,104,105,106]. The main dietary outcomes reported were a combination of macronutrients; carbohydrate, protein, fat, and alcohol (*n* = 66, 74%) [2,6,7,8,28,29,30,31,32,34,35,36,37,38,39,40,41,42,43,44,45,47,48,49,50,51,52,53,54,55,56,57,58,59,60,62,63,65,66,68,70,71,72,74,76,77,78,79,81,82,83,87,89,90,91,93,94,95,96,97,102,103,104,106]. Total energy intake was also frequently measured (*n* = 59, 66%) [2,6,7,20,28,29,30,31,32,34,35,37,38,39,40,41,42,43,48,49,50,51,53,54,55,56,57,58,59,60,65,66,68,70,71,72,74,75,76,77,79,81,82,88,89,90,91,93,94,95,96,97,100,102,103,104,105,106]. Alcohol was the most individually investigated source of energy (*n* = 19, 21%) and quantified mainly as grams consumed per day or as a total percentage of energy intake [6,20,25,34,43,50,60,61,62,70,71,75,79,82,83,96,97,98,102]. Micronutrients were reported in 46 (52%) [2,6,20,28,29,30,32,34,35,37,38,39,41,42,45,47,49,50,51,52,53,56,58,60,63,68,70,71,74,75,78,79,80,81,82,87,90,91,93,96,97,98,101,102,103] studies, and food groups reported 32 times (36%) [7,21,22,23,24,25,29,34,40,46,49,54,62,63,64,65,73,74,75,76,78,83,84,85,86,88,92,99,100,103,105]. Fibre intake was reported in 21 studies (24%) [8,31,34,35,37,38,45,50,54,56,65,71,72,75,81,90,94,96,97,101,103].

### 3.9. Comparison to Dietary Guidelines

Fifty three (60%) of the 89 studies reported a comparison with country-specific dietary guidelines [2,6,7,24,25,28,30,32,34,35,37,39,40,42,47,48,49,51,52,54,55,56,57,58,59,60,62,63,66,68,72,73,74,76,77,78,79,80,81,82,83,84,86,87,91,94,96,97,98,101,103,105]. Of these 39 (44%) compared with general dietary guidelines [7,24,25,28,35,37,40,42,49,51,54,55,57,58,62,63,66,68,70,72,73,74,76,77,78,80,82,83,84,86,87,91,94,96,97,98,101,103,105], one (1%) compared with sports nutrition guidelines [30]. Very few studies (*n* = 14 16%) specifically mentioned comparison with the military guidelines [2,6,32,34,37,39,47,48,52,56,59,60,79,81].

## 4. Discussion

The aim of this scoping review was to provide a broad overview of the dietary assessment methods used to assess the whole dietary intakes of military personnel and military veterans with fresh food access in a broad range of settings. The review identified 89 studies, of which 71 were conducted with active military personnel, with many published since 2010 indicating increased recognition of nutrition in the maintenance of health and military performance of personnel [109]. Most of the studies were based in the USA and were carried out in garrison settings and conducted less often in free-living military groups. Macronutrients were the most reported dietary outcome and the majority of studies reported a comparison with dietary counties. Despite the amount of research dedicated to developing military specific dietary guidelines, most studies did not compare against military specific guidelines.

Across all studies, FFQs were the most commonly used dietary intake method. FFQs have been shown to be a useful tool in the study of non-military populations, however, most FFQs have been designed for use in the general population, or for specific disease states [110]. Moreover, while most studies used validated tools, only two were reported to be validated in military populations. It is important to carry out such studies given the food lists within dietary assessment tools, such as FFQs, should be population-specific where possible. In 2009, Mullie et al. [71] developed a semi quantitative FFQ with 150 food items specific for military men, which was reported to reliably determine the dietary intake of military men. However, this tool was validated specifically within administrative military personnel and may not be valid in more intensive military settings [71]. Of the included studies, biomarkers used for validation purposes as objective measures of dietary intake were used in four studies; doubly-labelled water [37] and metabolites including alpha-carotene and beta-carotene [53,64,93]. This highlights the need for further research to validate self-reported dietary intake using objective measures rather than validating against other self-reported measures which have the same reporting bias. There was no apparent relationship between diet assessment method and study design.

Research has shown that between four to eight administrations of MPR, 24-hour recalls are required to minimise error in dietary intake data, with the inclusion of weekends or days off, to cover the change in intake [111,112,113]. Three of the studies included used the automated self-assessment 24-hour recall method at only one time point which is a limited reflection of usual dietary intake [111,113]. Wherever practicable, dietary research within military and military veteran populations should consider using the 24-hour MPR method over non-consecutive days to obtain data more likely to be reflective of habitual intake [114,115]. Recent research suggests combining methods of dietary intake collection is best to reduce the limitations of individual methodologies [113].

In this current review, total energy intake and macronutrients, including alcohol, were the most common dietary outcomes reported, with other aspects of dietary intake reported less often including diet quality. More specifically, alcohol consumption was the most commonly reported outcome component and was most often assessed through FFQs. This is an important area of research, given alcohol misuse is suspected to be attributed to increased stress exposure in military service, in particular those with post-traumatic stress disorder (PTSD) [116].

Results of this review show that army personnel (soldiers) is by far the most studied military population when it comes to the assessment of dietary intake. This could be contributed to army branches being the largest military branches in countries such as the USA where the majority of studies arise from, in addition to, Britain and Australia [117,118,119]. Armies are diverse and generally include groups of occupations from those directly involved in warfighting such as infantry, artillery, cavalry, and special forces, to those supporting front-line warfighters, such as Combat Engineers [14,120]. One of the most frequently studied sub-groups of the Army were those undertaking basic training or cadet training, which may be due to new recruits being placed in very physically demanding situations with increased nutrition requirements. The USA contributes the majority of research into the area of dietary intake amongst its military branches with 62% of studies originating from this country. This may be reflective of larger military groups, or higher access to military research funding.

When compared to veteran populations, the numbers of all actively serving personnel are much less by comparison [119]. For example, in the USA veteran numbers are reported at 20 million compared to around 400,000 currently serving in the US army [117]. In this current review, less than 25% of all studies included participants from this large veteran population indicating a large research gap for assessing and improving the diets of those in military veteran groups. The veteran population have differing nutritional needs and requirements post-discharge including transition to the civilian community, different accommodation environments, a more sedentary lifestyle in addition to high rates of mental illness, disability, and health conditions in general, thus it has been shown that the USA veteran population has an increased burden of disease when compared to non-veteran populations [121]. Given sup-optimal dietary intake is a leading contributor to disease, it is likely to contribute to a higher prevalence of ill health, or overweight and obesity rates of veterans, making this a research priority area [117,122]. A number of studies have demonstrated that military populations are experiencing a trend towards being increasingly obese, which mirrors the pattern among the general population [123]. A cross-sectional study undertaken by Breland et al. (2017) found the prevalence of obesity amongst veteran population in the USA was 41% (44% females, 41% males). Similarly, in a USA cohort study of the military, obesity among service members was 20%, and significantly higher among veterans (32%). A 2011 Australian Senate Estimates Brief titled “Obesity in Defence” stated that approximately 15% of Australian Defence Force personnel had a BMI in the obese range.

The current review also found a marked increase in studies investigating females only from 2000 onwards, which is important as 16% of the currently enlisted USA Military are female, an increase of 13% since voluntary military service commenced in the USA [124]. Studies regarding the dietary intake of female military members are notably less in numbers than those with a focus on male members, however, this has been increasing steadily post-2000. It has previously been reported by Goldzweig et al. (2006), that since 2000 there has been an increase in female military recruits who now make up 20% of total new recruits. This increase also coincides with the decision in 2013 by countries, including the USA and Australia, to open frontline combat roles to women [125,126]. The increased numbers of female military personnel has been recognised by the US Department of Veterans Affairs, and may explain the reason for the increase in focus on the specific dietary and health needs of female military recruits [127]. Another reason may be the unique nutrition requirements of females in the military. Many women in the US do not meet the Recommended Dietary Allowances of important nutrients such as calcium and iron, and in a military setting with increased physical training, these inadequate intakes could lead to long-term negative health outcomes [59].

This scoping review has several strengths including a comprehensive systematic search and review protocol, including a detailed data extraction process. Moreover, military experts were consulted at all stages in the development of this review to provide input to ensure data and reporting were accurate and relevant. Limitations include that only studies in English were included which may have limited the extent of military dietary data access. As such, non-English studies from countries such as China and Russia, which have large military spends and large military services, were not included. The current review reflects only those databases searched and may not reflect unpublished or other military specific journals not commonly available to researchers. Grey literature was also not searched, further limiting full access to published dietary intake data.

In conclusion, dietary intake in military settings is most commonly assessed via the FFQ and 24-hour recall methodologies, however not all tools used were specifically validated for military populations. Dietary intake reporting was mainly focused around overall individual energy intake, and the intake of macronutrients including carbohydrates, proteins, fats, and alcohol. Comparison to dietary guidelines was used in the majority of studies, however the comparison to specific military dietary guidelines is minimal.

## Figures and Tables

**Table 1:** Database search strategy.

**Military**
1 (military OR “military personnel” OR
2 (“defen?e force” OR “defen?e personnel”) OR
3 (army OR “air force” OR airforce OR navy OR “coast guard” OR naval” OR “armed forces” OR soldier* OR sailor* OR airmen OR airwomen) OR
4 (veteran* OR deployed)
5 AND (“English”)
6 AND
**Dietary**
1 (diet OR “diet records” OR “diet therapy” OR “dietary intake” OR
2 (diet* NEAR/5 (quality OR habit* OR behavio?r* OR pattern) OR
3 food OR
4 (food NEAR/5 (intake* OR habit* OR preference* OR consum*) OR
5 eating OR
6 nutrition OR
7 nutrient intake
AND (English)

**Table 2:** Included studies.

Author	Study Design	Country	Population	Type of Personnel	No. of Participants
**Military populations**					
Alexander et al. 1987 [28]	Cross-sectional	UK	Navy	Sailors	2311
Arsenault et al. 2000 [29]	Cross-sectional	USA	Army	Soldiers	50
Beals et al. 2015 [30]	Cross-sectional	USA	Army	Soldiers	439
Bedogni et al. 1999 [31]	Pre-Post	Italy	Army	Cadets	273
Belanger et al. 2016 [32]	Non-controlled trial	USA	Army	Soldiers	Baseline *n* = 136 Intervention *n* = 124
Bingham et al. 2012 [21]	Longitudinal Cohort	Finland	Army	Conscripts	604
Bingham et al. 2012 [33]	Longitudinal pre-post	Finland	Army	Conscripts	256
Bingham et al. 2009 [34]	Prospective cohort	Finland	Army	Conscripts	78
Botelho et al. 2014 [35]	Prospective cohort	Brazil	Army	Soldiers	92
Buffington et al. 2016 [36]	Randomised control trial (RCT)	USA	Air Force	Athletes/cadets	153
Carlson et al. 2013 [37]	Prospective cohort	USA	Army	Soldiers	53
Cline et al. 1998 [38]	Retrospective case-control	USA	Army	Soldiers	Cases *n* = 63 Control *n* = 78
Cline et al. 2000 [39]	RCT	USA	Army	Marines	Intervention *n* = 32 Control *n* = 31
Cole et al. 2018 [40]	Non-randomised control	USA	Army	Soldiers	688
Copp et al. 1991 [41]	Cross-sectional	USA	Air Force	Fighter pilots	30
Crombie et al. 2013 [7]	RCT with partial crossover design	USA	Army	Active duty soldiers	Baseline *n* = 602 Completed study *n* = 458
DeBolt et al. 1988 [42]	Observational	USA	Navy	SEAL Trainees	267
Deuster et al. 2003 [43]	Cross sectional	USA	Army	Rangers	38
Dwyer et al. 1981 [44]	Prospective cohort	Australia	Armed forces	Recruits	530
Edwards et al. 1987 [45]	Cross-sectional	England	Army	Recruits	Not reported (NR)
Eliasson et al. 2012 [46]	Cross-sectional	USA	Army	Soldiers	265
Etzion-Daniel et al. 2008 [47]	Prospective cohort	Israel	Army	Infantry and medics	Karakal females *n* = 92 Karakal males *n* = 33 Control females *n* = 48
Fallowfield et al. 2019 [48]	Pre and post - Cohort	UK	Army	Soldiers (marines, officers)	Pilot *n* = 37 Main study *n* = 98
Fiedler et al. 1999 [49]	Pre-Post	USA	Air Force	Recruits	Intervention *n* = 402 Control *n* = 422
Francois et al. 1997 [50]	Cross-sectional	France	Army	Soldiers	Strasbourg *n* = 344 Souge *n* = 1129
Frank et al. 2016 [51]	Prospective, longitudinal, cluster RCT	USA	Army	Soldiers	234
Friedl et al. 1995 [52]	Cross-sectional	USA	Army	Cadets	1979 *n* = 190 1990 *n* = 205
Gaffney-Stomberg et al. 2014 [53]	RCT	USA	Army	Recruits	168
Gambera et al. 1995 [54]	Randomised trial	USA	Air Force	Active duty air force	32 Exercise only *n* = 17 Exercise + diet *n* = 15
Hart et al. 1992 [55]	Cross-sectional	USA	Air Force	F-16 and F-15 pilots	*n* = 118
Herzman-Harari et al. 2013 [56]	Non-controlled trial 1-year follow-up	Israel	Army	Border Police (soldiers)	Baseline *n* = 44 2-mths *n* = 43 4-mths *n* = 38
Hilgenberg et al. 2016 [8]	Cross-sectional	Brazil	Air Force	Air Force cadets	166
Ismail et al. 1996 [57]	Observational Cross-sectional	Malaysia	Army	Soldiers	20
Jackson et al. 1983 [58]	Cross-sectional	USA	Army	Marine service men	2599
King et al. 1993 [59]	Cross-sectional	USA	Army	Soldiers	103
Klicka et al. 1996 [60]	Cross-sectional	USA	Army	Cadets	204
Kono et al. 1996 [61]	Cross-sectional	Japan	Army	Self-Defence forces	2062
Lutz et al. 2013 [62]	Pre-post	US	Army	Recruits	135
Lutz et al. 2017 [63]	Cross-sectional	US	Army and air force	Recruits	834
Lutz et al. 2019 [64]	Cross-sectional	USA	Navy	Marine recruits	380
Mathew et al. 2004 [65]	Case-control	US	Navy	Sailors	467
McAdam et al. 2018 [66]	Cross-sectional	USA	Army	Recruits	111
McClung et al. 2017 [67]	Cross-sectional	US	Army	Soldiers with rank of sergeant or below	131
Milne et al. 1980 [68]	Cross-sectional	US	Navy	Sailors	NR
Moran et al. 2012 [2]	Prospective cohort 4 and 6-mth follow-up	Israel	Army	Combat recruits	74
Mullie et al. 2012 [69]	Cross-sectional	Belgium	Army	Soldiers	1852
Mullie et al. 2015 [70]	Cross-sectional	Belgium	Army	Soldiers	1699
Mullie et al. 2009 [71]	Validation study	Belgium	Army	Soldiers	95
Mullie et al. 2016 [22]	Cross-sectional	Belgium	Army	Soldiers	7252
Mullie et al. 2012 [72]	Cross-sectional	Belgium	Army	Soldiers	1852
Mullie et al. 2009 [73]	Cross-sectional	Belgium	Army	Soldiers	1852
Nakayama et al. 2018 [74]	Cross-sectional	USA	Army, Air Force, Marines	Soldiers, air force, marines	401
Nkondjock et al. 2010 [75]	Cross-sectional	Central Africa	All services of the defence forces	Defence force members	541
Polikandrioti 2009 et al. [23]	Cross-sectional	Greece	Army	Recruits	1000
Purvis et al. 2013 [24]	Cross-sectional	US	Army	Soldiers	13,858
Rahmani et al. 2017 [76]	Cross-sectional	Iran	Army	Infantry soldiers	246
Ramsey et al. 2013 [6]	Cross-sectional s	US	Army	Soldiers	39
Royer et al. 2018 [77]	Cross-sectional Observational	USA	Army	Special Forces	215
Shams-White et al. 2019 [78]	Cross-sectional	USA	Army and Navy	Not specified	333
Singh et al. 1988 [79]	Cross-sectional	USA	Navy	Navy SEALS	16
Smith et al. 2013 [25]	Retrospective cohort	US	Army, navy, marine corps and air force	Military	15,747
Smoak et al. 1988 [80]	Cross-sectional	US	Navy	Recruits	16
Stark et al. 2008 [81]	Cross-sectional	Israel	Air Force	Pilots	31
Tharion et al. 2004 [82]	Observational cross-sectional	USA	Army	Special Forces and Support Personnel	45
Trent et al. 1988 [83]	Cross-sectional	US	Navy	Sailors	1013
Uglem et al. 2014 [84]	Non-randomised controlled trial 5-mth follow-up	Norway	Army	Recruits	479
Uglem et al. 2011 [85]	Cross-sectional	Norway	National Guard	Recruits	578
Uglem et al. 2013 [86]	Non-randomised controlled trial 5-mth follow-up	Norway	National Guard	Recruits	479
Versluis et al. 1973 [87]	Cross sectional	South Africa	Air Force	Air Force	51
Williamson et al. 2002 [88]	Cohort study	US	Army	Recruits	92
Young et al. 2017 [89]	RCT 10-week follow-up	US	Army	Military	71
Veterans					
Balali-Mood et al. 2014 [90]	Cross-sectional	Iran	Veterans	NR	110
Barboriak et al. 1978 [91]	Cohort observational	USA	Veterans	NR	51
Becerra et al. 2016 [92]	Cross-sectional	USA	Veterans	NR	11,011
Chapman et al. 1996 [93]	Prospective cohort	USA	Veterans	All	209
Ciubotaru et al. 2015 [94]	Double-blind placebo-RCT	USA	Veterans	Veterans	115
Gordon et al. 1985 [95]	Cross-sectional	USA	Veterans	Veterans	73
Hamirudin et al. 2016 [96]	Observational cross-sectional 3-mth follow-up	Australia	Veterans	Veterans	68
Kaye et al. 2015 [97]	Prospective Longitudinal	USA	Veterans	Veterans	533
Koutrakis et al. 2019 [98]	Cross-sectional	USA	Veterans	Veterans	183
Littman et al. 2015 [99]	Cross-sectional	US	Veterans	Veterans	150
Mehta et al. 2016 [100]	Longitudinal	US	Veterans	Veterans	839
Nosova et al. 2015 [101]	Cross-sectional	US	Veterans	Veterans	88
Park et al. 2009 [20]	Prospective cohort	US	Veterans	Veterans	586
Seddon et al. 2006 [102]	Prospective Cohort	US	Veterans	Veterans	1362 (681 twins)
Shahnazari et al. 2013 [103]	RCT 6-mth follow-up	US	Veterans	Veterans	60
Vidal et al. 2015 [104]	Case-control	US	Veterans	Veterans	430
Wang et al. 2017 [105]	Prospective, longitudinal cohort 21-year follow-up	US	Veterans	Veterans	983
Young et al. 1992 [106]	Cross-sectional	US	Veterans	Veterans	572

## Supplementary Materials

The following are available online at https://www.mdpi.com/2072-6643/12/3/769/s1, Table S1: Characteristics of included studies, Table S2: Study outcome characteristics
