# Supplementary material for: Dietary Assessment Methods in Military and Veteran Populations: A Scoping Review
Source: Nutrients. 2020 Mar 14;12(3):769. doi: 10.3390/nu12030769 (PMC7146105; doi:10.3390/nu12030769)
Supplement: Supplementary file 1 [file nutrients-12-00769-s001.zip › nutrients-720761-supplementary/Table S1 Nutrients.docx]

**Table S1.** Characteristics of included studies.

| **Author** | **Setting** | **Type of Personnel** | **Gender** | **Anthropometric data** | **Intervention Description** |
| --- | --- | --- | --- | --- | --- |
| **Military populations** |  |  |  |  |  |
| Alexander et al. 1987 ^(29)^ | Royal Navy ships (1 x aircraft carrier, 3 x destroyers and 2 x frigates) | Sailors | Male | NR | NR |
| Arsenault et al. 2000 ^(30)^ | Medical Officer Basic Course (Off-base in hotels or on-base with limited cooking facilities) | Soldiers | Female | Weight, BMI | NR |
| Beals et al. 2015 ^(33)^ | In garrison | Soldiers | Male  n= 372 Female n= 67 | Height, weight, BMI, %BF | NR |
| Bedogni et al. 1999 ^(35)^ | Italian Military Academy during IA or OA of food. IA - occurs 120 days a year during intensive physical training. | Cadets | Male | Weight, height, triceps skinfold, BMI, EER | NR |
| Belanger et al. 2016 ^(36)^ | Army installation | Soldiers | NR | BMI | Initial Military Training menu standards in non-trainee dining facilities |
| Bingham et al. 2012 ^(22)^ | Garrison canteens, Soldier's Home | Conscripts | Male | NR | Increased supply of healthy foods at garrison refectories and soldier's homes. 5 x workshops for staff to implement action plans |
| Bingham et al. 2012 ^(37)^ | Garrison canteens, Soldier's Home | Conscripts | Male | Weight, BMI, WC, muscle mass, fat mass, %BF | NA |
| Bingham et al. 2009 ^(38)^ | Southern Finland Armoured Brigade in garrison (Training 10 hours a day) | Conscripts | Male | Height, weight, BMI, WC | NA |
| Botelho et al. 2014 ^(39)^ | Northeast Brazilian Construction Unit | Soldiers | Male | Weight, height, BMI, BF % | NR |
| Buffington et al. 2016 ^(40)^ | Air Force Academy | Athletes/cadets | Female | DXA | 10 X CBT session COPE) - problem solving & coping skills. Three groups: (a) the combined energy balance educational intervention with COPE), (b) COPE alone (E2), and (c) a control group. |
| Carlson et al. 2013 ^(41)^ | 62nd Medical Brigade, Joint-Base Lewis-McChord | Soldiers | Male  Female | Height, weight, BMI, | NR |
| Cline et al. 1998 ^(44)^ | Military training installation | Soldiers | Female | Bone Density Evaluation, weight, height, BMI | NR |
| Cline et al. 2000 ^(45)^ | US Army | Marines | Male | Weight, %BF | Assigned to either a CHO drink group or a placebo during an 11-day field exercise. Fresh and semi-perishable rations were provided. |
| Cole et al. 2018 ^(46)^ | Military dining facilities | Soldiers | Male  Female | Weight, height, BMI | High-quality, nutrient-dense foods offered and incorporated into recipes. Reduction/ elimination of saturated fats in food preparation.  Placement of fruits, vegetables, whole grains, and other high-quality items in high-visibility areas. Nutrient-poor items inconvenient to acquire. |
| Copp et al. 1991 ^(47)^ | Tyndall Air Force Base Florida | Fighter pilots | Male | NR | NR |
| Crombie et al. 2013 ^(8)^ | 10 DFACs (military dining facilities) on Fort Bragg, NC | Active duty soldiers | Male  n = 562 Female  n =54 | Height, weight, BMI | Increased availability of fruit, vegetables, and wholegrains. Decreased availability of discretionary foods. Increase in low fat protein and dairy portions. Staff educated on traffic light system. |
| DeBolt et al. 1988 ^(48)^ | US SEALs at Training School | SEAL Trainees | Male | Height, weight, BMI, BF% | NR |
| Deuster et al. 2003 ^(49)^ | Fort Benning, Army Barracks Georgia | Rangers | NR | Weight, height, BF%, | NR |
| Dwyer et al. 1981 ^(50)^ | Armed Forces Base (Adelaide) | Recruits | Male | BF, height, weight, BMI | NR |
| Edwards et al. 1987 ^(51)^ | Military units British Army | Recruits | Male  Female | NR | NR |
| Eliasson et al. 2012 ^(52)^ | Pennsylvania Army National Guard | Soldiers | Male  Female | Height, weight, BMI | NR |
| Etzion-Daniel et al. 2008 ^(53)^ | Karakal Unit | Infantry & medics | Male  Female | Height, weight, BMI. BF%, LBM | NR |
| Fallowfield et al 2019 ^(54)^ | Military camps | Soldiers (marines, officers) | Male | Body mass, height and body composition | NR |
| Fiedler et al. 1999 ^(55)^ | Lackland Airforce Base | Recruits | Male  Female | Height, weight, BMI | Eating from Heart Healthy menu. Control: eating from regular Air Force menu. |
| Francois et al. 1997 ^(56)^ | Military College Strasbourg + Parachutist corps Souge | Soldiers | NR | NR | NR |
| Frank et al. 2016 ^(57)^ | 2 x Combat arm brigades pre and post deployment | Soldiers | Male  Female | Height, weight, WC, BF, BMI, bone density | Telehealth coaching Control: One-time nutrition and fitness education |
| Friedl et al. 1995 ^(58)^ | US Military Academy | Cadets | Male  Female | Height, weight, BF%, Body circumferences | NR |
| Gaffney-Stomberg et al. 2014 ^(59)^ | Military dining facility | Recruits | Male  Female | Height, weight, BMI, BF% | High calcium and Vitamin D snack bars.  Control: Placebo snack bars = no added calcium and Vitamin D. Both groups instructed to have 2 bars per day. |
| Gambera et al. 1995 ^(60)^ | McClellan Air Force Base medical clinic | Active duty air force | Male  Female | Height, weight, BMI | All completed 90-day exercise program 3 x week. Exercise-plus-diet group received weekly individualised counselling from a dietitian - focus on US dietary guidelines and food pyramid |
| Hart et al. 1992 ^(63)^ | US Air Force Bases | F-16 & F-15 pilots | Male | Height, weight | NR |
| Herzman-Harari et al. 2013 ^(64)^ | Basic training | Border Police (soldiers) | Female | Height, weight, BMI | 15 sessions by Dietitian on a range of topics from macronutrients, food groups, food guide pyramid, use of food labels.  Signs were posted in the living quarters with slogans on healthy nutrition and the soldiers received handouts. |
| Hilgenberg et al. 2016 ^(9)^ | Brazilian Air Force Academy | Air Force cadets | Male n=147 Female n=19 | Height, weight, BMI, WC, BF% | NR |
| Ismail et al. 1996 ^(65)^ | Malaysian Base Camp | Soldiers | NR | Weight, height, BMI, BF%, LNM | NR |
| Jackson et al. 1983 ^(66)^ | US Military bases | Marine service  men | Male | NR | NR |
| King et al. 1993 ^(68)^ | Two in field and two in dining halls | Soldiers | Female | Height, weight, BF | NR |
| Klicka et al. 1996 ^(69)^ | US Military Academy, | Cadets | Male n=118 Female n=86 | NR | NR |
| Kono et al. 1996 ^(70)^ | Self-Defence Forces | Self-Defence forces | Male | Weight, Height, BMI, WHR | NR |
| Lutz et al 2013 ^(73)^ | Basic combat training | Recruits | Male  Female | NR | NR |
| Lutz et al 2017 ^(74)^ | 2 × military training bases | Recruits | Male  Female | BMI | NR |
| Lutz et al. 2019  ^(75)^ | Military camps | Marine recruits | Male  Female | Height, weight, BMI, body fat % | NR |
| Mathew et al 2004 ^(76)^ | Navy Medical Centre | Sailors | Male  Female | BMI | NR |
| McAdam et al. 2018 ^(77)^ | Military camps | Recruits | Male | Height, weight | NR |
| McClung et al 2017 ^(78)^ | 1 x military assessment camp US Army's Ranger Selection and Assessment Program | Soldiers with rank of sergeant or below | Male | Height, weight and BMI | NR |
| Milne et al 1980 ^(80)^ | Naval air station | Sailors | Male  Female | NR | NR |
| Moran et al 2012 ^(2)^ | 1 x elite combat unit in Israel defence force | Combat recruits | Male | Height, weight and BMI, BF % | NR |
| Mullie et al 2012 ^(81)^ | Community-based | Soldiers | Male | BMI | NR |
| Mullie et al 2015 ^(82)^ | Community-based | Soldiers | Male | BMI | NR |
| Mullie et al 2009 ^(83)^ | Military administrative centre | Soldiers | Male | NR | NR |
| Mullie et al 2016 ^(23)^ | Community-based | Soldiers | Male  Female | BMI, weight | NR |
| Mullie et al 2012 ^(84)^ | Community-based | Soldiers | Male | BMI | NR |
| Mullie et al 2009 ^(85)^ | Community-based | Soldiers | Male | BMI | NR |
| Nakayama et al. 2018 ^(86)^ | Varied military settings | Soldiers, air force, marines | Male  Female | BMI, height, weight | NR |
| Nkondjock et al 2010 ^(87)^ | 8 x military institutions | Defence force members | Male  Female | BMI, waist circumference | NR |
| Polikandrioti 2009 et al ^(24)^ | Hospital-based | Recruits | Male | BMI, own beliefs regarding the body weight | NR |
| Purvis et al 2013 ^(25)^ | Community-based | Soldiers | Male  Female | BMI, WC | NR |
| Rahmani et al. 2017 ^(89)^ | Army barracks | Infantry soldiers | Male | Weight, height, BMI | NR |
| Ramsey et al 2013 ^(7)^ | 2 x military bases | Soldiers | Male  Female | Height, Weight, BMI, WC | NR |
| Royer et al. 2018 ^(90)^ | NR | Special Forces | Male | Height, weight, body composition | NR |
| Shams-White et al. 2019 ^(93)^ | Army barracks & 1 naval site | Not specified | Male  Female | NR | NR |
| Singh et al. 1988 ^(94)^ | US Navy | Navy SEALS | Male | Height, weight, BF % | NR |
| Smith et al 2013 ^(26)^ | Community-based | Military | Male  Female | BMI | NR |
| Smoak et al 1988 ^(95)^ | Naval base | Recruits | Male | NR | NR |
| Stark et al 2008 ^(96)^ | Air force headquarters | Pilots | Male | Weight, height, BMI | NR |
| Tharion et al. 2004 ^(97)^ | Military dining facility | Special Forces & Support Personnel | Male | Height, weight, DXA | NR |
| Trent et al 1988 ^(98)^ | 9 x navy ships | Sailors | Male | % body fat | NR |
| Uglem et al 2014 ^(99)^ | 2 x Military recruit training centres | Recruits | Male | NR | Increased availability of vegetables and wholegrain bread. Nutritional education re. health benefits of diets rich in vegetables, fruit and whole grains. 2 project workers were available for 2 days every sixth week to answer questions. |
| Uglem et al 2011 ^(100)^ | Military recruit training centre | Recruits | Male | BMI | NR |
| Uglem et al 2013 ^(101)^ | 3 x military recruit camps | Recruits | Male | NR | Increased availability of vegetables and wholegrain bread. Nutritional education re. health benefits of diets rich in vegetables, fruit and whole grains. 2 project workers were available for 2 days every sixth week to answer questions. |
| Versluis et al 1973 ^(102)^ | Community-based | Air Force | Male | NR | NR |
| Williamson et al 2002 ^(105)^ | Military base – basic combat training | Recruits | Male  Female | Weight, Height, BMI % overweight | NR |
| Young et al 2017 ^(107)^ | Active-duty military population living in the Baton Rouge, LA | Military | Male  Female | Weight, Height, BMI | Experimental-moderate group: diet low in n-6 fatty acids and a high proportion n-3 fatty acids.  Experimental-high group: same diet as moderate but also consumed a smoothie with 1000 mg of n-3 fatty acids per 200 ml.  Control group and moderate group received a placebo smoothie. |
| **Veterans** |  |  |  |  |  |
| Balali-Mood et al. 2014 ^(31)^ | Off-base | NR | Male | Height, weight (%BF, BMI) | NR |
| Barboriak et al. 1978 ^(32)^ | Veterans Administration Domiciliary | NR | Male | NR | NR |
| Becerra et al. 2016 ^(34)^ | California Health Interview Survey | NR | Male n = 10196 Female n = 815 | BMI | NR |
| Chapman et al. 1996 ^(42)^ | Seattle VA Medical Centre | All | Male | Weight, height, BMI, WC | NR |
| Ciubotaru et al. 2015 ^(43)^ | Veterans Administration | Veterans | Male | BMI, total BF %, Android BF % | Group 1: individuals with normal glucose tolerance.  Group 2: individuals with impaired glucose tolerance and impaired fasting glucose. Microbiota composition analysed in stool collected at end of intervention. Compared against Group 1 and 2 as well as lowest and highest quartiles of energy and fat, hemoglobinA1c, Vitamin D. |
| Gordon et al. 1985 ^(61)^ | Denver Veterans Geriatric Ambulatory Care Clinic | Veterans | Male  n=72  Female  n=1 | Height, weight | NR |
| Hamirudin et al. 2016 ^(62)^ | In-home | Veterans | Male n=32 Female n=36 | Height, weight, BMI | Home based dietetic care. |
| Kaye et al. 2015 ^(67)^ | Veterans Affairs Dental Longitudinal | Veterans | Male | Height, weight, BMI | NR |
| Koutrakis et al. 2019 ^(71)^ | Veterans Affairs Medical Centre | Veterans | Male  Female | Height, weight, BMI, DXA | NR |
| Littman et al 2015 ^(72)^ | Community-based | Veterans | Male  Female | BMI | NR |
| Mehta et al 2016 ^(79)^ | VA Normative Aging Study | Veterans | Male | Height, % Obesity (BMI >30 kg/m2) | NR |
| Nosova et al 2015 ^(88)^ | Outpatient clinic | Veterans | Male  Female | Height; Weight; BMI; Waist-to-hip ratio | NR |
| Park et al 2009 ^(21)^ | Community-based | Veterans | Male | BMI | NR |
| Seddon et al 2006 ^(91)^ | Community-based | Veterans | Male | BMI | NR |
| Shahnazari et al 2013 ^(92)^ | Community-based | Veterans | Male  Female | Body weight BMI | 60-min individualised nutrition education with 15-min wellness coaching over 6 mths.  Control: 60-min individualised nutrition education session from dietitian student. |
| Vidal et al 2015 ^(103)^ | Medical centre | Veterans | Male | BMI | NR |
| Wang et al 2017 ^(104)^ | Unclear | Veterans | Male | BMI | NR |
| Young et al 1992 ^(106)^ | Unclear | Veterans | Male | Weight, BMI, ; | NR |
