# Supplementary material for: Dietary Assessment Methods in Military and Veteran Populations: A Scoping Review
Source: Nutrients. 2020 Mar 14;12(3):769. doi: 10.3390/nu12030769 (PMC7146105; doi:10.3390/nu12030769)
Supplement: Supplementary file 1 [file nutrients-12-00769-s001.zip › nutrients-720761-supplementary/Table S2 Nutrients.docx]

**Table S2.** Study outcome characteristics.

| **Author** | **Dietary intake assessment method** | **Validated/Standardised tool** | **Duration** | **Dietary intake reported** |
| --- | --- | --- | --- | --- |
| **Military Populations** |  |  |  |  |
| Alexander et al. 1987 ^(29)^ | Analysis of all daily food offered (with wastage factors) + daily confectionary sales | No | 14 days | Energy, macronutrients, micronutrients, cholesterol, fatty acids |
| Arsenault et al. 2000 ^(30)^ | 7-days of self-reported food intakes on blank food record cards. Interview with dietitian to review completeness and accuracy. | Yes | 7 days | Frequency of foods consumed, energy, macronutrients, micronutrients, food groups |
| Beals et al. 2015 ^(33)^ | Nutrition History Questionnaire and 24-hour recall | Yes | 24 hours | Meals per day, eating frequency, energy, macronutrients, micronutrients |
| Bedogni et al. 1999 ^(35)^ | Sampling of meals after food service (food served & then frozen for later analysis). Foods purchased from store were analysed for energy content. | NR | 2 randomly selected weeks. 5 days of meal sampling | Energy, carbohydrate, fat, protein, fibre, whole foods |
| Belanger et al. 2016 ^(36)^ | Observation of participant’s food selection and a digital photograph used to measure food selections and nutrient intake at one lunch meal, participant’s plate waste weighed at completion of meal. | Yes | 4 weeks. 2 days of data collection - 1 at baseline 1 3-weeks post IMT implementation | Energy, macronutrients, micronutrients, nutrition quality |
| Bingham et al. 2012 ^(22)^ | Food choices & 36-item FFQ - reported as days in previous week - adapted from Finnish population studies. Diet measured in first week of service & again at 6-months | Yes | Control: questionnaires at 1, 2 and 6 months. Intervention: questionnaires at 1 week, 2 and 6 months | Items per week of breads, fruit, vegetables and discretionary items |
| Bingham et al. 2012 ^(37)^ | Food choices & 36-item FFQ - reported as days in previous week - adapted from Finnish population studies. Diet measured in first week of service & again at 6 months | Yes | 6 months | Items per week of breads, fruit, vegetables and discretionary items |
| Bingham et al. 2009 ^(38)^ | Food diaries. Sub study 1 - Food recorded weekdays and weekends. Sub study 2 - encampment days | Yes | Sub study 1 - 4 days.  Sub study 2 - 3 days | Energy, macronutrients, fatty acids, alcohol, fibre, micronutrients, food groups |
| Botelho et al. 2014 ^(39)^ | Preparation of all meals and snacks observed via the Technical Preparation Files method and their nutritional composition was determined. Meals weighed and served to participants. Waste weight collected. Not completed for breakfast or snacks | Yes | 3 days | Energy, macronutrients, fibre, sodium |
| Buffington et al. 2016 ^(40)^ | 24-hour recall | Yes | 12 weeks | Carbohydrate, protein, total fat, saturated fat |
| Carlson et al. 2013 ^(41)^ | Block FFQ | Yes | 12 months | Energy, macronutrients, calcium, magnesium, sodium, phosphorus, vitamin K, vitamin D |
| Cline et al. 1998 ^(44)^ | FFQ - including recall from teenage years | Yes | NR | Energy, macronutrients, micronutrients, fibre |
| Cline et al. 2000 ^(45)^ | Trained estimators recorded amount served and amount returned by participants. Dietary log cards used by individuals and were reviewed by trained data collectors | Yes | 10 days | Energy, protein, fat, carbohydrate, cholesterol, micronutrients |
| Cole et al. 2018 ^(46)^ | Demographic survey, food photography, customer satisfaction survey | Yes | 12 months | Energy, macronutrients, fruit, vegetables, dairy, grains, protein foods, oils/fats, added sugar |
| Copp et al. 1991^(47)^ | 24-hr recall and Food frequency checklist | Yes | 5 months | Energy, percent of military recommended dietary allowances, protein, micronutrients, percent of total energy from carbohydrate, protein and fat |
| Crombie et al. 2013 ^(8)^ | Digital photograph before and after meal and processed through food portion estimation software (staff and participants trained for photography of plates) | Yes | 12 months | Energy, macronutrients, total fat, discretionary fats, wholegrains, refined grains, fruit and vegetables |
| DeBolt et al. 1988 ^(48)^ | 1-day diet record. Recorded on Web/Thurs/Fri by randomisation | Yes | NR | Energy, macronutrients, cholesterol, sodium, potassium, micronutrients |
| Deuster et al. 2003 ^(49)^ | Food Frequency portion of Block National Cancer Institute Health Habits and History Questionnaire | Yes | NR | Energy, macronutrients, alcohol |
| Dwyer et al. 1981 ^(50)^ | FFQ | Yes | 2 years | Energy, total fat, saturated fat, monounsaturated fat, polyunsaturated fat, carbohydrate, protein, cholesterol, energy, macronutrients |
| Edwards et al. 1987 ^(51)^ | Analysis of the total food issued to kitchens with allowances for cooking losses. Pre- and post-stock check (weighed and checked) for total unit consumption | NR | 3 months | Energy, fat, sugar, sodium, fibre |
| Eliasson et al. 2012 ^(52)^ | Mediterranean Diet Questionnaire | Yes | 6 months | Meat, fruit and vegetables |
| Etzion-Daniel et al. 2008 ^(53)^ | FFQ | Yes | 16 weeks | Protein, micronutrients |
| Fallowfield et al 2019 ^(54)^ | 4-day food diary. Accuracy assessed using the James & Schofield human energy requirement equation for BMR calculation. | Yes | 2 years | Energy, macronutrients |
| Fiedler et al. 1999 ^(55)^ | Health Habits & History Questionnaire | Yes | 6 weeks | Energy, protein, fat, legumes, calcium, breads and cereals, fruit and vegetables |
| Francois et al. 1997 ^(56)^ | Food consumption survey with weighed records. Drink machine consumption measured. Food waste measured | Yes | 3 days | Energy, whole foods, alcohol, macronutrients, calcium, phosphorus, magnesium, iron, fibre |
| Frank et al. 2016 ^(57)^ | Block FFQ - 110 items | Yes | 12-months | Energy, macronutrients, micronutrients |
| Friedl et al. 1995 ^(58)^ | 7-day food diary (33 males reviewed by dietitian interview), food preparation and portion weights monitored. Same conducted for 5-day record in 1979 (no dietitian interviews) | Yes | 7 days (1990)  5 days (1979) | Energy, macronutrients, cholesterol, sodium, saturated fat, polyunsaturated fat |
| Gaffney-Stomberg et al. 2014 ^(59)^ | Self-administered Block FFQ under the supervision of Dietitians. Habitual dietary intakes for the 3 months prior to coming to BCT (pre-) and during BCT (post-) | Yes | 3 months | Energy, macronutrients, calcium, Vitamin D |
| Gambera et al. 1995 ^(60)^ | FFQ. Exercise-plus-diet group also completed 3-day food intake records each week | Yes | 12 weeks | Energy, macronutrients, cholesterol, fibre, food groups |
| Hart et al. 1992 ^(63)^ | 3-day dietary record portions checked by exercise physiologist | Yes | 3 days | Energy, macronutrients, alcohol, caffeine |
| Herzman-Harari et al. 2013 ^(64)^ | FFQ | Yes | 4 months | Energy, macronutrients, water, fibre, micronutrients |
| Hilgenberg et al. 2016 ^(9)^ | 3-day food diary (non-consecutive, 1 x weekend day) | Yes | 7 months | Protein, carbohydrate, lipids, saturated fat, monounsaturated fat, polyunsaturated fat, cholesterol, fibre |
| Ismail et al. 1996 ^(65)^ | 7-day weighed food record. Food consumed outside of mess recorded by participants | Yes | 5 weeks | Energy, protein, fat, carbohydrate |
| Jackson et al. 1983 ^(66)^ | 14-day diet records obtained by interviews at 3-4 day intervals. Interviewers had nutrition and dietetics degrees | Yes | 14 days | Energy, protein, iron, folate, vitamin C, B12, B6, copper, zinc |
| King et al. 1993 ^(68)^ | NR | NR | Field: Hawaii (44 days), Bolivia (15 days).  Dining halls: West point (5 days), Fort Jackson (7 days) | Energy, macronutrients |
| Klicka et al. 1996 ^(69)^ | Trained recipe data collectors recorded information about the ingredients, preparation and portion weights of foods served in the Cadet Mess.  Participants recorded their intakes of food, beverages, nutritional supplements, salt, and water for 7 consecutive days on food record forms. Trained dietitians interviewed cadets daily to review and verify food record entries. | Yes | 7 days | Energy, macronutrients, saturated fat, monounsaturated fat, polyunsaturated fat, cholesterol, micronutrients, alcohol |
| Kono et al. 1996 ^(70)^ | Dietary questions asked about the consumption frequency and amount of six food items (brewed coffee, instant coffee, green tea, milk, rice, and soy paste soup) and the consumption frequency of 10 food items (bread for breakfast, cheese, pickles, raw vegetables, fruits, raw fish, soy sauce-cooked fish, broiled fish, meats, and garlic) on average in the past year. | NR | 2 years | Rice, soy paste soup, pickles, bread, cheese, raw vegetables, fruit, fish, meat, garlic, milk, brewed coffee, instant coffee, alcohol, green tea |
| Lutz et al 2013 ^(73)^ | Block FFQ 2005 - Semi-quantitative FFQ | Yes | 9 to 10 weeks | HEI, sodium, saturated fat, solid fat, alcohol, added sugar, oils, milk, fruit, grains, meat and beans, vegetables |
| Lutz et al 2017 ^(74)^ | Block FFQ 2005 - Semi-quantitative FFQ | Yes | June 2012–March 2014 | Fruit, vegetables, protein, grains, dairy, fatty acid, sodium, solid fat, added sugar, chicken, meat, eggs, soy, fish and seafood, alcohol,  saturated fat, monounsaturated fat, polyunsaturated fat |
| Lutz et al. 2019 ^(75)^ | Block FFQ 2014 | Yes | 18 months | Alpha-carotene, beta-carotene, folate, fruit, vegetables |
| Mathew et al 2004 ^(76)^ | FFQ | Yes | 2 years | Energy, red meat, fat, fruit, vegetables, fibre |
| McAdam et al. 2018 ^(77)^ | Dietary intake recorded after each meal on 3 non-consecutive days. Food menus from the cafeteria-style dining facility used to create meal specific diet logs containing meal specific food items and serving sizes for each food item. | NR | 14 weeks | Energy, fat, protein, carbohydrate, energy |
| McClung et al 2017 ^(78)^ | Digital food photography of breakfast lunch and dinner. Total daily energy expenditure (TDEE) estimated using doubly labelled water. | Yes | 5-days | Energy, protein, carbohydrate, fat, calcium, fibre, iron, magnesium, phosphorous, potassium, selenium, sodium, zinc, Vitamin A, B6, B12, C, D, E, folate, niacin, riboflavin, thiamine |
| Milne et al 1980 ^(80)^ | Analysis of food offered minus plate waste and food returned to the kitchen. Sub group of men also had food and beverage consumption collected for 14 days using a diary-interview technique. | Yes | 2 weeks | Energy, protein, copper, zinc, manganese. |
| Moran et al 2012 ^(2)^ | FFQ (126 food items divided into nine food groups) developed for the Israeli population by Ben-Gurion University | Yes | 6 months | Energy, protein, carbohydrates, fat, iron, folate, calcium, zinc, magnesium, Vitamin D, B6 and B12 |
| Mullie et al 2012 ^(81)^ | Semi-quantitative FFQ | Yes | NR | Dietary patterns, SSB’s and ASB’s |
| Mullie et al 2015 ^(82)^ | Semi-quantitative FFQ | Yes | NR | Energy, dietary patterns, protein, carbohydrate, total fat, saturated fat, alcohol, added sugar, sodium, calcium, iron, HEI, MDS |
| Mullie et al 2009 ^(83)^ | Semi-quantitative FFQ and 4-day dietary records | Yes | 2 weeks | Energy, protein, carbohydrate, fibre, fat, saturated fat, monounsaturated fat, polyunsaturated fat, sodium, potassium, calcium, iron, cholesterol, alcohol, vitamin A, B1, B2, C |
| Mullie et al 2016 ^(23)^ | Online survey of daily food intake and weekly frequency of SSB consumption | No | 2 weeks | Breakfast, SSB’s, fruit, vegetables, meat |
| Mullie et al 2012 ^(84)^ | Semi-quantitative FFQ | Yes | NR | Energy, protein, carbohydrate, total fat, saturated fat, monounsaturated fat, polyunsaturated fat, sugar, fibre, low-fat foods |
| Mullie et al 2009 ^(85)^ | Semi-quantitative FFQ | Yes | NR | Fortified margarines, fermented dairy, nuts, black tea, red wine, fruit, vegetables, fatty fish |
| Nakayama et al. 2018 ^(86)^ | Two versions of FFQ: Block 2005 and Block 2014.  The Block 2005 was used for the Army and Air Force and includes ˜110 food and beverage items to estimate average daily nutrient intake over the past 3 mo.  The Block 2014 was used for all the Marine recruits. It includes ˜127 food and beverage items and asks individuals to report intake over the past 6 months. | Yes | NR | Energy, calcium, potassium, protein, carbohydrate, fat, added sugar, oils/fats, dairy, fruit, vegetables, grains, legumes, eggs, lean meats, seafood, nuts and seeds, chicken |
| Nkondjock et al 2010 ^(87)^ | FFQ | Yes | NR | Energy, fibre, calcium, fruit, vegetables, oils/fats, alcohol, fish and seafood, fruit juices, vegetable juices, rice, herbs and spices, pasta, milk, soft drinks, honey and jam, meat, cereals, tea, coffee, bread, dietary patterns, supplements, |
| Polikandrioti 2009 et al ^(24)^ | Self-report questionnaire | No | NR | White and red meat, bread, sports drinks, snacks, desserts, fast food, easy to prepare foods, fruit |
| Purvis et al 2013 ^(25)^ | 5-item Healthy Eating Score (HES-5). | NR | 2 weeks | Fruit, vegetables, whole grains, dairy, fish |
| Rahmani et al. 2017 ^(89)^ | Semi-quantitative FFQ | Yes (not for military) | 1 month | Energy, macronutrients, fatty acids, meat, grains, fruit, vegetables, nuts, legumes, trans fat, polyunsaturated fat |
| Ramsey et al 2013 ^(7)^ | National Cancer Institute DHQ - an FFQ | Yes | NR | Energy, protein, carbohydrate, total fat, saturated fat, monounsaturated fat, polyunsaturated fat, fibre, cholesterol, vitamin A, B12, C, E, folate, calcium, iron, potassium, sodium, alcohol, caffeine |
| Royer et al. 2018 ^(90)^ | 24 h dietary recall | Yes | 1 day | Energy, macronutrients, fat |
| Shams-White et al. 2019 ^(93)^ | FFQ | Yes | 2.5 years | Protein, total fat, saturated fat, sugar, sodium, fruit, vegetables, grains, dairy, HEI, food groups |
| Singh et al. 1988 ^(94)^ | 3-day diet records (consecutive) | Yes | 3 days | Energy, macronutrients, alcohol, micronutrients |
| Smith et al 2013 ^(26)^ | Subset of questions in the 2005 Department of Defence Health Related Behaviours Survey. | No |  | Alcohol, fruit, vegetables, wholegrains, low fat dairy, regular fat dairy, lean protein, snack foods, sweets, fast food |
| Smoak et al 1988 ^(95)^ | 1-day diet records collected twice over 5-day period and reviewed by a dietitian. | Yes | 10 days | Sodium, potassium, calcium, phosphorous, magnesium, iron, zinc, Vitamin A, C, E, B6, B12, thiamine, riboflavin, niacin. |
| Stark et al 2008 ^(96)^ | Individual interviews using a questionnaire developed by the Israel Centre for Disease Control and the Ministry of Health. Questionnaire included a validated 24-hour food consumption questionnaire. | Yes | 6 months | Energy, protein, carbohydrate, total fat, saturated fat, monounsaturated fat, polyunsaturated fat, cholesterol, calcium, iron, zinc, magnesium, sodium, Vitamin C, A, E and B12, folic acid, fibre |
| Tharion et al. 2004 ^(97)^ | Record of food items and amount on tray before and after eating.  Intake of each food item was calculated as the difference between amount taken and amount returned. Volunteers recorded all food consumed outside the dining facility, including dietary supplements and weekend consumption when the dining facility was closed. | Yes | 9-days | Energy, macronutrients, alcohol, micronutrients |
| Trent et al 1988 ^(98)^ | Lifestyle questionnaire | No | NR | Discretionary salt, lean meat, high-fat meat, high-fat dairy, low-fat dairy, saturated fat, polyunsaturated fat, eggs, sugar, fruit, vegetables, high-fibre bread and cereals, caffeine, alcohol |
| Uglem et al 2014 ^(99)^ | Validated food diary developed at University of Oslo Nutrition department | Yes | 5 months. | Fruit, vegetables, semi wholegrain bread |
| Uglem et al 2011 ^(100)^ | 4-day food diary developed at Department of Nutrition, University of Oslo | Yes | Not specifically reported. Surveys collected in both Jan 2004 and July 2004 (2 different study groups). | Fruit, vegetables, breakfast cereals, semi wholegrain bread, SSB’s |
| Uglem et al 2013 ^(101)^ | Validated food diary developed at University of Oslo Nutrition department | Yes | 5 months | Fruit, vegetables, juice, potatoes, white bread, semi wholegrain bread |
| Versluis et al 1973 ^(102)^ | 2 types of dietary intake questionaries - 1 × filled in with interviewer, 1 x filled in at home | No | NR | Protein, fat, saturated fat, unsaturated fat, carbohydrate, calcium, phosphorus, iron, sodium, potassium, Vitamin A, micronutrients |
| Williamson et al 2002 ^(105)^ | Digital Photography - photos of food trays taken before and after eating, analysed by research assistants | No | 8 weeks | Energy, fruit, vegetables, grains, milk, meat, fat, added sugar |
| Young et al 2017 ^(107)^ | Analysis of all food offered and any additional items consumed that were not provided by study staff. | No | Aug 2012 to Nov 2013 | Energy, carbohydrate, protein, total fat, saturated fat, monounsaturated fat, omega 3 and 6 fatty acids. |
| **Veterans** |  |  |  |  |
| Balali-Mood et al. 2014 ^(31)^ | FFQ | Yes | NR | Energy, macronutrients, micronutrients, starch, fibre, fatty acids |
| Barboriak et al. 1978 ^(32)^ | Intake observed over 5 days. Measured served meals (3 meals/day, 2 choices at each meal. Observation by trained observers of food eaten at meals times + weighed leftovers. Reporting and questioning of between meal food and beverage intake. Voluntary reporting of alcohol intake | Yes | 5 days | Energy, macronutrients, micronutrients, fatty acids |
| Becerra et al. 2016 ^(34)^ | Not clearly stated - past week consumption of fast foods, sodas, fruits and vegetables | NR | 1 week | Servings per week of fast food, soda, fruits and vegetables |
| Chapman et al. 1996 ^(42)^ | 24-hour recall in structured interviews & validated by FFQ. Conducted by dietitians | Yes | 3 years | Energy, macronutrients, micronutrients, cholesterol |
| Ciubotaru et al. 2015 ^(43)^ | 2 × 24hr recalls. A subgroup of 40 subjects provided 2–3 sets of dietary data to evaluate for dietary intake consistency. | Yes | 12 months | Energy, macronutrients, fibre |
| Gordon et al. 1985 ^(61)^ | 24-hr intake of total calories. Assisted by a dietitian. Clarification with household member when required. Catalogue of foods eaten weekly | Yes | 10 months | Energy, carbohydrate, fat, protein |
| Hamirudin et al. 2016 ^(62)^ | Diet history & FFQ conducted by dietitian | Yes | 3 months | Energy, protein, carbohydrate, fat, alcohol, water, fibre, micronutrients |
| Kaye et al. 2015 ^(67)^ | Harvard University FFQs + Daily servings of each food group were compared with the kcal-specific DASH recommendations. | Yes | 17 years | Energy, protein, total fat, saturated fat, alcohol, sucrose, fructose, lactose, sugars, calcium, phosphorous, fibre, starch, soft drinks |
| Koutrakis et al. 2019 ^(71)^ | Harvard University FFQs + health questionnaire based on the American Thoracic Society Questionnaire. | Yes (not for military) | 3 years | Vitamin D, calcium, alcohol |
| Littman et al 2015 ^(72)^ | FFQ | Yes | June to Nov 2011 | Fast food, snacks, fruit and vegetables, soda/sweet tea, beans, chicken, fish, chips, crackers, non-low-fat desserts and other sweets, margarine/butter/meat fat |
| Mehta et al 2016 ^(79)^ | Semi-quantitative FFQ adapted from the questionnaire used in the Nurses’ Health Study. | Yes | May 1992 and October 2008 | Energy, fruit, vegetables, fibre, hot and cold cereal, vitamin C, caffeine, omega 3 fatty acids, dark fish, cold cults, flavanones |
| Nosova et al 2015 ^(88)^ | Block FFQ 2005 - Semi-quantitative FFQ | Yes | NR | Omega-3 fatty acids, cholesterol, sodium, potassium, calcium, magnesium, fibre |
| Park et al 2009 ^(21)^ | Semi-quantitative FFQ adapted from the Nurses’ Health Study | Yes | Nov 2000 and June 2007 | Energy, alcohol, fruit, vegetables, carotenoids, dark fish, omega-3 fatty acids, Vitamin C |
| Seddon et al 2006 ^(91)^ | FFQ | Yes | NR | Energy, protein, alcohol, fish, beta-carotene, zinc, vitamin C and E, multivitamins, omega 3 fatty acids |
| Shahnazari et al 2013 ^(92)^ | Block FFQ 2005 - Semi-quantitative FFQ. | Yes | May 2010 and Jan 2011 | Energy, carbohydrate, total fat, saturated fat, trans fat, cholesterol, sodium, fibre, sugar, protein, calcium, vitamin D, iron, fruit, vegetables, breads and cereals, meat, fish, beans, eggs, dairy, fats and oils, sweets, SSB’s, wholegrains |
| Vidal et al 2015 ^(103)^ | Willett FFQ | Yes | Jan 2007 to June 2012 | Energy, carbohydrate, protein, fat, fibre, glycaemic index |
| Wang et al 2017 ^(104)^ | Semi-quantitative FFQ | Yes | 1987–2008 | Energy, prudent pattern score, western pattern score, red/processed meat, seafood, chicken, eggs, butter, fries, high-fat dairy, fruit, vegetables, refined grains, wholegrains |
|  |  |  |  |  |
| Young et al 1992 ^(106)^ | Semi-quantitative FFQ | Yes | Feb 1987 to June 1989 | Energy, protein, fat, carbohydrate |
